# Ephrin B1 and B2 Mediate Cedar Virus Entry into Egyptian Fruit Bat Cells

**DOI:** 10.3390/v17040573

**Published:** 2025-04-16

**Authors:** Lea Lenhard, Martin Müller, Sandra Diederich, Lisa Loerzer, Virginia Friedrichs, Bernd Köllner, Stefan Finke, Anca Dorhoi, Gang Pei

**Affiliations:** 1 Institute of Immunology, Friedrich-Loeffler-Institut, Federal Research Institute for Animal Health, 17493 Greifswald, Germany; lea.lenhard@fli.de (L.L.); lisa.loerzer@fli.de (L.L.); bernd.koellner@fli.de (B.K.); anca.dorhoi@fli.de (A.D.); 2 Institute of Molecular Virology and Cell Biology, Friedrich-Loeffler-Institut, Federal Research Institute for Animal Health, 17493 Greifswald, Germany; mamue122@hhu.de (M.M.); stefan.finke@fli.de (S.F.); 3 Institute of Novel and Emerging Infectious Diseases, Friedrich-Loeffler-Institut, Federal Research Institute for Animal Health, 17493 Greifswald, Germany; sandra.diederich@fli.de; 4 Institute of Diagnostic Virology, Friedrich-Loeffler-Institut, Federal Research Institute for Animal Health, 17493 Greifswald, Germany; virginia.friedrichs@fli.de; 5 Faculty of Mathematics and Natural Sciences, University of Greifswald, 17489 Greifswald, Germany

**Keywords:** Cedar virus, henipaviruses, ephrins, viral entry, receptor, *Rousettus aegyptiacus*

## Abstract

Cedar virus (CedV), closely related to the Hendra and Nipah viruses, is a novel *Henipavirus* that was originally isolated from flying foxes in Australia in 2012. Although its glycoprotein G exhibits relatively low sequence similarity with its counterparts of the Hendra and Nipah viruses, CedV also uses ephrin receptors, i.e., ephrins B1, B2, A2 and A5, to enters human cells. Nevertheless, the entry mechanism of CedV into bat cells remains unexplored. Considering that *Rousettus aegyptiacus* (Egyptian Rousette bat, ERB) is postulated to be a reservoir host for henipaviruses, we aim to reveal the receptors utilized by CedV to enable its entry into ERB cells. To this end, we cloned the class A and B ephrins of ERB and generated CHO-K1 cells stably expressing individual ephrins. We also developed a lentivirus-based pseudovirus system containing the firefly luciferase reporter. Assessment of the luciferase activity in cells expressing single ephrins demonstrated that the ERB ephrin B1 and B2 mediated CedV pseudovirus entry. Further, we generated a recombinant CedV expressing the fluorescent protein TurboFP635 (rCedV-nTurbo635). By performing high-content microscopy and flow cytometry, we unveiled that, in addition to ephrin B1 and B2, ephrin A5 was also able to mediate rCedV-nTurbo635 entry, although to a much lesser extent. In contrast to human ephrin A2, ERB ephrin A2 failed to mediate rCedV-nTurbo635 entry. Finally, we generated ERB epithelial cells with ephrin B1 and/or ephrin B2 knockdown (KD). The entry of rCedV-nTurbo635 into ERB epithelial cells was drastically impaired by ephrin B1/B2 KD, validating the importance of ephrin B1 and B2 in its entry. Altogether, we conclude that CedV primarily employs ERB ephrin B1, B2 and, possibly, A5 for its entry into ERB cells.

## 1. Introduction

Hendra virus (HeV) and Nipah virus (NiV) are negative-sense single-stranded RNA viruses belonging to the genus *Henipavirus* within the family *Paramyxoviridae* [1]. They are classified as biosafety level-4 pathogens due to their high pathogenicity and ability of cross-species transmission [2,3,4]. HeV was first discovered in 1994 in Brisbane, Australia, as the causative agent of fatal respiratory and neurological disease in horses and close-contact humans [5,6,7]. NiV, which is closely related to HeV, first emerged in Malaysia and Singapore between 1998 and 1999 and caused similar symptoms in pigs and humans [8,9,10]. Since then, several NiV outbreaks have been documented in Bangladesh and India, with mortality rates of 40–70% [9,11,12,13]. Multiple fruit bat species within the genus *Pteropus* have been identified as reservoir hosts for both viruses [14,15,16,17,18,19]. Although HeV and NiV outbreaks are assumed to be limited to Australia and South(-East) Asia, they are also associated with the distribution of Pteropus bats, and other henipaviruses and henipavirus-like viruses are widely distributed in different bat species worldwide, which may pose a great threat to global public health [9]. Accumulating serological and molecular evidence has revealed the association of known henipaviruses and novel henipavirus-like viruses with non-*Pteropus* fruit bats, i.e., *Eidolon dupreanum*, *Eidolon helvum*, *Rousettus madagascariensis* and *Rousettus aegyptiacus* in African countries and Madagascar [20,21,22,23,24]. Egyptian Rousette bats (*Rousettus aegyptiacus*, ERB) are widely distributed across Sub-Saharan Africa, Egypt, Cyprus, Southwest Asia and parts of the Middle East [25,26,27,28,29]. Numerous henipavirus-related sequences have been identified in samples from ERB in African countries [30,31,32], supporting that ERB may be reservoir hosts for henipaviruses or henipavirus-like viruses [30]. Thus, investigating henipaviral infection in ERB cells is an important step to understand the susceptibility of this potential reservoir to henipaviruses.

Recently an increasing number of novel henipaviruses have been discovered in bats, shrews and rodents, such as Ghanaian bat virus [23], Mojiang virus [33], Langya virus [34] and Angavokely virus [35]. Some of them, such as Cedar virus (CedV) and Langya virus, have been successfully isolated [36,37,38]. CedV originates from urine samples of *Pteropus* bats in Australia, and it shows high similarity to HeV in its genome composition [37]. Regardless of this similarity, CedV is considerably less virulent since it lacks the pathogenicity factors V and W, which result from RNA editing of the P gene mRNA [37,39,40,41]. The P, V and W proteins of HeV and NiV efficiently inhibit interferon (IFN) signaling in human cells by blocking STAT1/2 activation [42,43,44,45]. Additionally, the paramyxoviral V proteins inhibit MDA5-, as well as RIG-I-dependent IFN induction and antiviral responses [46,47]. The NiV W protein also suppresses NF-κB activation and NF-κB-dependent proinflammatory responses [48]. In contrast to the efficient inhibition of IFN signaling by P proteins of HeV and NiV, the CedV P protein exhibits impaired inhibition of STAT1/2 activation in human cells [42,43,44,45,49]. Consequently, CedV infection induces strong IFN responses in vitro, leading to reduced viral replication compared to NiV and HeV [37,39,50,51,52]. Furthermore, guinea pigs, hamsters and ferrets challenged with CedV demonstrate transient virus replication and seroconversion without developing clinical disease [37,39]. Thus, CedV lacks critical virulence factors and does not pose major risks for humans. Given its close genetic relatedness to HeV and NiV, CedV has been proposed as a novel platform for henipavirus vaccine development [40].

Since host and tissue tropism of henipaviruses is associated with receptor usage, the research on CedV entry into host cells is of great importance [38,53,54,55,56]. HeV and NiV entry into human cells is mediated by their receptors ephrin B2 and B3 [57,58,59,60]. Ephrins are divided into two groups, the ephrin A class, containing ephrin A1 to A5, and the ephrin B class, including ephrin B1 to B3. The A ephrins are associated with the plasma membrane via a glycosylphosphatidylinositol (GPI) anchor [61,62], while the B ephrins are embedded in the cell membrane through their transmembrane domains [61,62]. Upon binding to the G-H loop of ephrin B2/B3 [2,63,64], NiV/HeV glycoprotein G tetramers undergo a conformational change, leading to exposure of the G stalk C-terminal domain. This, in turn, results in interaction with the fusion protein F and its conformational change, thus triggering cell and virus membrane fusion and, subsequently, the release of the viral genome into the cell [2,56,65,66].

Although CedV G protein only shares 30% amino acid sequence similarity with the HeV/NiV G protein, CedV also employs ephrins for its entry into human cells [37,40,67]. In contrast to HeV/NiV, CedV mainly uses ephrins B1 and B2, as well as ephrins A2 and A5 to enter human cells [67]. However, the receptors mediating CedV entry into bat cells remain unknown. Therefore, in this study, we generated a CedV pseudovirus and the recombinant fluorescent Cedar virus (rCedV-nTurbo635), and we also performed high-content microscopy imaging and flow cytometry assays with the aim to elucidate ERB ephrins, which is used by CedV for its entry. Importantly, we generated ERB nose epithelial cells with ephrin B1 and/or B2 knockdown (KD) to validate our findings. Collectively, we conclude that CedV mainly employs ERB ephrins B1, B2 and, likely, A5 for its entry into ERB cells.

## 2. Material and Methods

### 2.1. Cell Culture

To obtain an epithelial cell line from ERB, the nasal epithelium was digested with 0.25% trypsin for 12 h at 37 °C and cell dissociation was terminated by adding serum-containing IMDM/F-12 (1:1) medium (Gibco, Waltham, MA, USA). Subsequently, cells were seeded in 6-well plates and transfected with a plasmid expressing Simian virus 40 large T (SV40LT) antigen using Lipofectamine™ 2000 (ThermoFisher, Waltham, MA, USA) according to the manufacturer’s instructions. To obtain single-cell clones of generated immortalized cells, limiting dilution was performed. The generated stable cell line was designated as RaNep. For ERB lung endothelial cells, lung pieces containing blood vessels were digested with trypsin/EDTA, and the dissociated cells were cultured with a commercial endothelial cell growth medium (PromoCell, Heidelberg, Germany) or a homemade medium with the same composition. Then, enriched endothelial cells were characterized by the positive staining of von Willebrand factor. Chinese hamster ovary cell line CHO-K1, human embryonic kidney cell line HEK293T and baby hamster kidney cell line BHK-21 were obtained from the Cellbank at Friedrich-Loeffler-Institut. CHO-K1 and RaNep were cultivated in Ham’s F12/Iscove’s modified Dulbecco’s medium IMDM (1:1), containing 10% (1:10) fetal calf serum (FCS) (P30-3302, PAN™ Biotech, Aidenbach, Germany), 1% Penicillin-Streptomycin (100× solution, VWR, Leuven, Belgium) and 2 mM of L-Glutamine (X0550-100, Biowest, Nuaille, France). HEK293T were maintained in Dulbecco’s modified Eagle’s medium (DMEM) (high glucose, 4.5 g/L), which was supplemented with 10% (1:10) FCS, 1% Penicillin-Streptomycin, 2 mM of L-Glutamine, 1% non-essential amino acids (NEAA, 100×, Gibco, London, UK) and 1 mM of sodium pyruvate (Gibco, London, UK). BHK-21 were cultivated in minimum essential medium (MEM) offset with Earle’s and Hanks’ balanced salts, NEAA with addition of 10% FCS, 1% Penicillin-Streptomycin and 2 mM of L-Glutamine. All cells were kept at 37 °C and 5% CO_2_ in a humidified incubator.

### 2.2. Plasmids, Antibodies and Reagents

pMD2.G (#12259), psPAX2 (#12260), pLKO.1 (#10878), pcDNA-FLAG (#20011) and p3xFLAG-ATF6 (#11975) were acquired from Addgene. For easy sorting of knockdown cells, GFP and the internal ribosomal entry site (IRES) were inserted upstream of the puromycin-resistant gene of the pLKO.1 vector to create a modified vector, which is designated as pLKO.1-GFP hereinafter. pHAGE2-CMV-Luciferase-ZsGreen was kindly provided by Max Josef Kellner (Institute of Molecular Biotechnology, Vienna, Austria). Ephrin A2, A3, A4, B1 and B3 were synthesized at Eurofins Genomics, with the nucleotide sequences optimized according to human codon usage. Ephrin A1, A5 and B2 were amplified from the cDNA of cells from ERB. Ephrins A3 and A5 were subcloned into p3xFLAG-ATF6 vector containing three consecutive FLAG tags at the N-terminus. The other ephrins were cloned into a pcDNA-FLAG vector with the tag at the C-terminus. CedV G and M sequences were synthesized at Eurofins Genomics and subcloned into pEGFP-C1 vector (Clontech Laboratories, San Jose, CA, USA). The generation of pCAGGS-CedV F with a C-terminal HA-tag was conducted as described before [68]. All plasmids were verified by Sanger sequencing.

The antibodies used in this study include the following: anti-β-Actin (66009-1-Ig, Proteintech, Rosemont, IL, USA); anti-DYKDDDDK tag (20543-1-AP, rabbit polyclonal, Proteintech, USA); anti-DYKDDDDK tag (66008-4-Ig, mouse IgG2b, Proteintech, USA); Alexa Fluor 488 conjugated anti-mouse-IgG (AB_2534069, Invitrogen, Carlsbad, CA, USA); BV-421 conjugated anti-mouse-IgG (Poly4053, BioLegend, San Diego, CA, USA); and anti-ephrin B2 antibody (26533-1-AP, Proteintech, USA).

Polyethylenimine (PEI) (#24314-2, Polysciences, Warrington, PA, USA); Geneticin G418—Sulfat 50 mg/mL (A6798, AppliChem, Darmstadt, Germany); Puromycin dihydrochloride (CAYM13884-25, VWR International, Leuven, Belgium); DAPI (D9542, Sigma-Aldrich, Taufkirchen, Germany); ProLong™Diamond Antifade Mountant with DAPI (P36962, Invitrogen, USA); Paraformaldehyde 32% (#15714, Electron Microscopy Sciences, Hatfield, PA, USA); Saponin (232-462-6, Sigma Aldrich, St. Louis, MO, USA); UltraPure ^TM^ bovine serum albumin (BSA) (AM2616, ThermoFisher Scientific, Lithuania); LunaScript^®^ RT SuperMix Kit ((E3010, New England BioLabs, Ipswich, MA, USA); Luna Universal qPCR Master Mix (M3003, New England BioLabs, USA); True-Nuclear™ Transcription Factor Buffer Set (#424401, BioLegend, USA); Zombie UV™ Fixable Viability Kit (#423107, BioLegend, USA); and Dimethylsulfoxide (DMSO) (D12345, Thermo Fisher Scientific Life Technologies Corporation, Carlsbad, CA, USA) were purchased as indicated.

### 2.3. Generation of CHO-K1 Cells Stably Expressing ERB Ephrins (CHO-EFN)

CHO-K1 cells were transfected in 12-well plates using polyethylenimine (PEI) as the transfection reagent according to the established protocol [69] with 2 μg of plasmid encoding for the various ephrins. For ephrin A3 and A5, 4 μg of these plasmids were used due to their lower expression levels. At 24 h post transfection, Geneticin G418 (A6798, AppliChem, Darmstadt, Germany) was added to the cell culture medium at a final concentration of 300 μg/mL for several weeks until cells reached confluency. In subsequent cultivation, G418 concentration was maintained at the same level.

### 2.4. Recombinant Cedar Virus (rCedV-nTurbo635) Generation and Infection

For the generation of fluorescence reporter expressing rCedV-nTurbo635, the TurboFP635 red fluorescence protein coding sequence [70] was N-terminally fused to three copies of SV40 derived nuclear localization signal coding sequence, and it was inserted as an extra cistron in a CedV cDNA full-length clone between the CedV P and M genes. rCedV-nTurbo635 was generated from the cDNA by transfection into BSR T7/5 cells [71], as has been described earlier [72]. The resultant virus was amplified and titrated on BSR T7/5 cells. rCedV-nTurbo635 infections were performed in DMEM containing 10% FCS. After 90 min incubation, the virus inoculum was removed and replaced by a cell culture growth medium. For MOCK infection, PBS was added to the infection medium.

### 2.5. High-Content Imaging Analysis of CedV Entry

CHO-K1 cells stably expressing ERB ephrin B1, B2 and B3 were seeded in 96-well plates at 1 × 10^4^ cells/well for 24 h and then infected with rCedV-nTurbo635 at MOI 1, 2 and 5. CHO-K1 cells stably expressing ERB ephrin A1–A5 were seeded in the same manner and given the low expression levels, and they were then re-transfected on Day 2 with 0.3 μg of plasmid per well and infected with rCedV-nTurbo635 on Day 3. All infections were performed in duplicates, and three independent experiments were performed. CHO-K1 cells stably expressing pcDNA-FLAG as negative controls and BHK-21 cells as positive controls were included in each experiment, as BHK-21 cells are deficient in IFN signaling and CedV can replicate to high titers in these cells [39]. Plates were fixed in 4% PFA (*w*/*v* in PBS) for 20 min, washed with PBS and, subsequently, stained for 10 min with DAPI. They were then diluted 1:1000 in PBS containing 0.1% saponin to decrease the exposure time in automated plate scanning. After two more wash steps, plates were imaged and analyzed using the CellInsight™ CX7 Pro HCS-platform. Briefly, the cell mask was generated based on DAPI nuclei staining and, subsequently, filtered by size, shape and brightness to obtain the valid primary objects. In the second channel (ex. 561 nm), another mask detecting the red fluorescence adjacent to the valid primary objects was created to identify the virus infected cells. For analysis of the infection rate, the parameter “Average intensity of channel 2” was used and only “highly” positive events, as determined by the aforementioned controls, were interpreted as infected cells. Thresholds of fluorescent intensities were determined based on the signal from negative controls and positive controls.

### 2.6. Immunofluorescence

CHO-K1 cells stably expressing ephrins were seeded at 1 × 10^5^ cells on glass coverslips in 12-well plates and infected at MOI 2. At 24 h post infection (p.i.), cells were fixed with 4% PFA for 20 min at 4 °C. For immunofluorescence staining, the protocol was modified from the one published by Fiegl et al. [73]. Coverslips were incubated with the mouse anti-DYKDDDDK-tag antibody (1:1250) for 1 h at RT, washed three times with PBS, followed by incubation with Alexa Fluor 488 conjugated anti-mouse IgG antibody (1:400). After three wash steps, coverslips were fixed on slides using ProLong™Diamond Antifade Mountant with DAPI (P36962, Invitrogen, USA). Slides were incubated overnight, in the dark at 4 °C in a wet chamber and, on the next day, dried in the dark before fixing them in place with clear nail polish. Images were taken using a Nikon Eclipse Ti microscope, and editing was conducted in ImageJ/Fiji (1.48v; Java 1.6.0_20[64-bit]).

### 2.7. Generation of Lentiviral shRNA Plasmids

shRNA oligos were designed using the Broad Institute’s Genetic Perturbation Platform (https://portals.broadinstitute.org/gpp/public/seq/search, accessed on 10 July 2023). For ephrin B1 and B2, three targets each were synthesized by metabion GmbH and cloned into the pLK0.1-GFP vector. The targeting sequences against ERB ephrin B1 and B2 are included in Table 1. All plasmids were validated by Sanger sequencing.

### 2.8. Lentivirus Production and Transduction of RaNep Cells

HEK293T cells were seeded in a 12-well plate at 2 × 10^5^ cells/well and incubated for 24 h. For transfection PEI with 1.0 μg of psPAX2, 1.0 μg of pMD2.G and 2 μg of the shRNA constructs targeting ephrin B1/B2 was used, as described before [74]. As a negative control, scrambled shRNA was used. Then, 6 h after transfection, the medium was changed to DMEM containing 20% FCS, and, 48 h after transfection, supernatants containing lentiviruses were collected and centrifuged at 350× *g* for 10 min to remove cells and debris.

To transduce RaNep cells, 8.0 × 10^4^ cells per well were seeded in a 12-well plate for 24 h, and 1 mL of the supernatants containing corresponding lentiviruses were added into each well of RaNep cells. Then, 2 μg/mL polybrene and cyclosporin A (5 μg/mL) were included to enhance transduction efficiency. At 72 h post transduction, Puromycin (1 μg/mL) was added to select the transduced RaNep cells. After around 2–3 weeks of selection, positive colonies formed and were selected for expansion.

### 2.9. mRNA Extraction, cDNA Synthesis and qPCR

RNA extraction was performed according to the protocol published by Chomczynski et al. [75]. RNA concentrations were determined using the NanoDrop One (Thermo Scientific, USA). For reverse transcription, the LunaScript^®^ RT SuperMix Kit was used according to the manufacturer’s instructions. After transcription, cDNA was diluted with 30 μL of RNAse-free water and then stored at −20 °C until further use. The primers were designed using the PrimerQuest tool (Integrated DNA Technologies, Coralville, IA, USA) to meet the following requirements: primer lengths of around 17–30 bp, optimal melting temperature at 61–62 °C, GC content of 40–55%, and amplicon length of 100–250 bp. Primer pairs were examined with the OligoAnalyzer tool (Integrated DNA Technologies, USA) to exclude the presence of homo- and/or heterodimer or hairpin formation. PCR reactions were carried out with 100 ng of cDNA and performed with the QuantStudio 6 Flex Real-Time PCR System (ThermoFisher, USA). PCR products were sequenced to confirm their specificity. Generally, qPCR was performed according to the protocol established before [76]. For the PCR reaction, Luna Universal qPCR Master Mix was employed with an addition of 1% DMSO. Optimal annealing was achieved at 62 °C.

### 2.10. Species Identification by Cytochrome c Oxidase Subunit I (COI) PCR

To confirm the Egyptian Rousette bat origin of the RaNep knockdown cells, species identification PCR was performed according to the protocol established by Cooper et al. [77]. To design COI primers for Tamarin monkey (*Saguinus oedipus*) and ERB (*Rousettus aegyptiacus*), COI barcoding sequences for these species were retrieved from the BOLD system (https://www.boldsystems.org/, accessed on 30 January 2023). The primers used in this study are listed in Table 2.

### 2.11. Flow Cytometry Analysis of rCedV-nTurbo635 Entry

CHO-K1 cells stably expressing ERB ephrins were seeded in a 6-well plate at 3 × 10^5^ cells per well and incubated overnight. Then, cells were re-transfected with 5 μg of corresponding ephrin plasmids. Medium was changed to 2% serum 3 h after transfection, and rCedV-nTurbo635 infection at MOI 5 was performed an additional 24 h later. One well of BHK-21 infected at MOI 0.1 served as a positive control for rCedV-nTurbo635 infection. At 48 h, p.i. cells were carefully washed with PBS and detached with 0.5 mL of 0.5% trypsin per well. Cells were resuspended in 1 mL of cell culture medium and pelleted down at 350× *g* for 5 min at 4 °C. Subsequently, cells were washed with FACS Buffer (Ca^2+^/Mg^2+^ free PBS offset with 0.1% BSA and 0.1% Sodium azide) and stained with Zombie UV™ Fixable Viability Kit (423107, BioLegend, USA), which was diluted 1:500 in 100 μL of FACS buffer for 20 min at 4 °C. After washing, cells were fixed in fixation buffer (True-Nuclear™ Transcription Factor Buffer Set (424401, BioLegend, USA)) for 30 min at room temperature in the dark and then permeabilized by washing the samples twice in permeabilization buffer (True-Nuclear™ Transcription Factor Buffer Set (424401, BioLegend, USA)). In order to stain the FLAG-tagged ephrins, samples were incubated with 100 μL of permeabilization buffer containing the anti-DYKDDDDK tag antibody (1:1000 dilution, Proteintech, Rosemont, IL, USA) for 20 min at 4 °C. Cells were washed once more and then stained with the BV-421 conjugated anti-mouse IgG antibody at 1:50 dilution for 20 min at 4 °C. After two more washes, cells were resuspended in 500 μL of FACS buffer, filtered through a 150 μm nylon filter sheet (510–9528, VWR, Leuven, Belgium) and measured at the BD FACSymphony A3™. RaNep ephrin knockdown cells were treated similarly after infection at MOI 1; however, they were only stained with the viability dye (ZombieUV) and resuspended in 800 μL of total volume before measurement. Data analysis was conducted using FlowJo™ (v_10.9.0) and Graph Pad Prism™ (10.1.2).

### 2.12. SDS-PAGE and Western Blot

CHO-K1 cells expressing ERB ephrins were seeded in 6-well plates (0.5 × 10^6^ cells per well) and, after 24 h, re-transfected with their respective ephrins to increase expression levels. At 24 h post transfection, cells were lysed with 300 μL of 1× RIPA buffer supplemented with Pierce™ phosphatase inhibitor mini tablets (A32955, Thermo Scientific, USA) on ice for 10 min. Afterward, samples were centrifuged at 17,000× *g* for 10 min at 4 °C, and the supernatants were then boiled with Laemmli buffer at 95 °C for 5 min. After this, samples were loaded onto 12.5% SDS gels, and those were then run at 65 V for 30 min and an additional 1 h at 120 V. Proteins were transferred onto nitrocellulose membranes using a semi-dry blotter (Bio-Rad, Feldkirchen, Germany). Then, the membranes were blocked with 5% skim milk in PBST (PBS + 0.05% Tween-20) for 2 h and incubated with primary antibodies at 4 °C overnight. The secondary antibody incubation was performed at room temperature for 1 h. Membranes were developed using the SuperSignal™ West Femto Kit (ThermoFisher, USA) and imaged with the ChemiDoc MP system (Bio-Rad Laboratories, Germany). ACTB/β-actin was used as the loading control.

### 2.13. Generation of Lentivirus-Based Pseudotyped CedV Particles

To optimize the protocol of pseudotyped CedV generation, 8 × 10^6^ HEK293T cells were seeded into a 10 cm dish overnight to be transfected with 5 μg of PsPax, 2.5 of μg pHAGE-CMV-luciferase-ZsGreen and different amounts of CedV G and F (3, 4, 5, 6 and 7 μg). To investigate the effect of the matrix and nucleocapsid protein on pseudovirus production, 1.5 μg of plasmids containing CedV M or N were also included for transfection. In subsequent experiments, we downsized to a 6-well plate to save reagents. Briefly, 3 × 10^6^ HEK293T cells/well were seeded in a 6-well plate and 2 μg of PsPax2, 2 μg of pHAGE-CMV-luciferase-ZsGreen, 2 μg of CedV G and 2 μg of CedV F were utilized for transfection. The same amounts of NiV G and F were used as a positive control. At 6 h post transfection, the medium was replaced with fresh DMEM medium containing 20% FCS. The supernatants containing pseudotyped particles were collected at 24 h after transfection and were centrifuged at 500× *g* for 3 min to remove cell debris.

### 2.14. Pseudotyped CedV Infection Assay

To reveal the specific ERB ephrins mediating CedV pseudovirus entry, 1 × 10^5^ CHO-K1 cells per well in a 24-well plate were transfected with 2 μg of ephrin A1-A5 and B1-B3 plasmids. Next, 24 h after transfection, the medium was removed and 1 mL of supernatants containing pseudotyped particles was added into each well. After 48 h of incubation, cell lysates were collected for luciferase measurement in a Tecan spark spectrophotometer.

### 2.15. Statistical Data Analysis

To determine the statistical significance between groups, ordinary one-way analysis of variance (ANOVA) was performed on the pseudovirus data. To determine the significance of the rCedV-nTurbo635 infection rate in flow cytometry, one-way ANOVA with Dunnett’s multiple comparisons test was performed. The remaining data were analyzed using a Kruskal–Wallis test without Dunn’s correction as the data were distributed non-parametrically. All statistical analyses were performed using GraphPad Prism 10.1.2. Statistically significant differences were defined as *p* < 0.05 (*), *p* < 0.01 (**), *p* < 0.001 (***) and *p* < 0.0001 (****).

## 3. Results

### 3.1. CedV Pseudotyped Particles Enter CHO-K1 Cells Expressing ERB Ephrins B1 or B2

**Figure 1:**
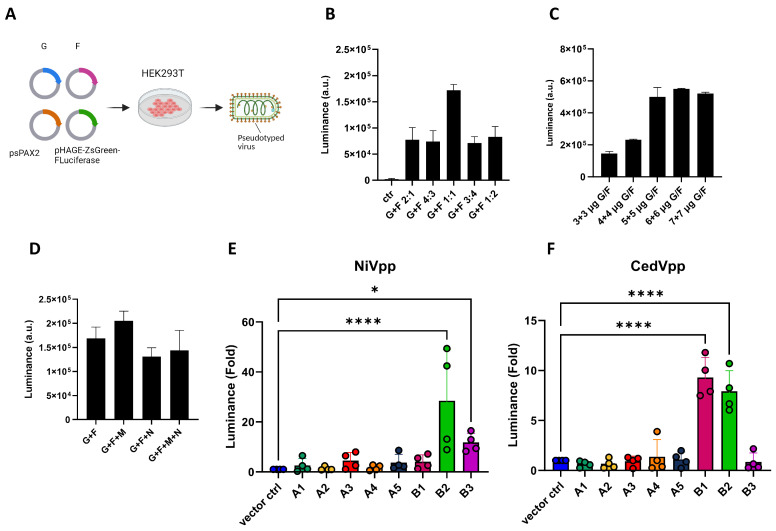
The expression of ERB ephrin B1 or B2 mediates the entry of Cedar pseudotyped particles. (**A**) Scheme of pseudovirus generation. For the generation of pseudotyped particles, HEK293T cells were transfected with the transfer plasmid (pHAGE-ZsGreen-luciferase), packaging plasmid (psPAX2) together with CedV/NiV G and F. The supernatant containing the pseudotyped particles was collected at 24 h post transfection and centrifuged, and the supernatants were then directly used for infection of CHO-K1 transiently expressing ERB ephrins. The images were created with Biorender.com. (**B**,**D**) Optimization of CedVpp generation by transfection of different ratios of G/F plasmids as indicated (**B**), or in different amounts (**C**) or in the presence of M and/or N plasmids (**D**). Bars show the mean ±SEM. The pseudovirus particles were added onto BHK21 cells to examine the yield under different conditions. (**E**,**F**) Entry of pseudotyped particles expressing NiV G/F (**E**) or CedV G/F (**F**) into CHO-K1 cells transfected with plasmids encoding for ERB ephrins. *Y*-axis shows the fold change in luciferase induction normalized to negative control (vector ctrl.). Bars show the mean ± SEM. Statistical analysis was performed with one-way ANOVA. *p* < 0.05 (*), *p* < 0.01 and *p* < 0.0001 (****).

CedV is reported to use ephrin B1, B2, A2 and A5 as its receptors to enter human cells [40,67]. However, although ephrins A1-A5 and B1-B3 of the Egyptian Rousette bat (ERB) are similar to their human counterparts concerning their amino acid sequences (Figure S1), whether the same ephrins mediate CedV entry into ERB cells remains to be addressed. Therefore, we aim to elucidate the ephrins enabling the entry of CedV into ERB cells. Since the original wildtype CedV isolate is restricted to the BSL4 area, we first generated CedV pseudotyped viral particles (CedVpp) using a lentivirus-based pseudovirus system to investigate CedV entry. The pseudovirus particles were generated by transfecting HEK293T cells with a lentiviral transfer plasmid encoding the firefly luciferase, psPAX2 as the packaging plasmid and CedV G and F (Figure 1A). As an internal control to validate the method, we included NiV pseudovirus particles (NiVpp) in the same luciferase entry assay. First, we tested different ratios of CedV G to CedV F and their impact on pseudovirus yield, thereby identifying a ratio of 1:1 as superior to other ratios (Figure 1B). NiV matrix protein (M) is essential for virus assembly and budding by connecting the ribonucleoprotein complex to the glycoproteins F and G [78,79,80]. However, the incorporation of CedV M or nucleocapsid protein (N) into the pseudovirus system did not lead to significant improvement of CedVpp yield (Figure 1C). To assess which ERB ephrins mediate CedVpp entry, CHO-K1 cells, which do not express endogenous ephrins [81], were transfected with individual ERB ephrins. Subsequently, these cells were incubated with the pseudotyped particles, and pseudovirus entry was evaluated by measuring the firefly luciferase activity in cell lysates. In accordance with the literature [58,59], NiVpp entry was mediated by ERB ephrins B2 and B3 in our assay (Figure 1D), validating the reliability of our pseudovirus system. Compared to CHO-K1 cells transfected with the empty vector, significant CedVpp entry was only detected in cells expressing ephrin B1 and B2, but not in cells expressing other ephrins (Figure 1E). This finding is consistent with previous publications on human ephrin B1 and B2 [40,67,82]. However, no significant entry of CedVpp was observed in cells expressing either ephrin A2 or A5.

### 3.2. Recombinant Cedar Virus Utilizes ERB Ephrins B1, B2 and A5 for Its Entry

**Figure 2:**
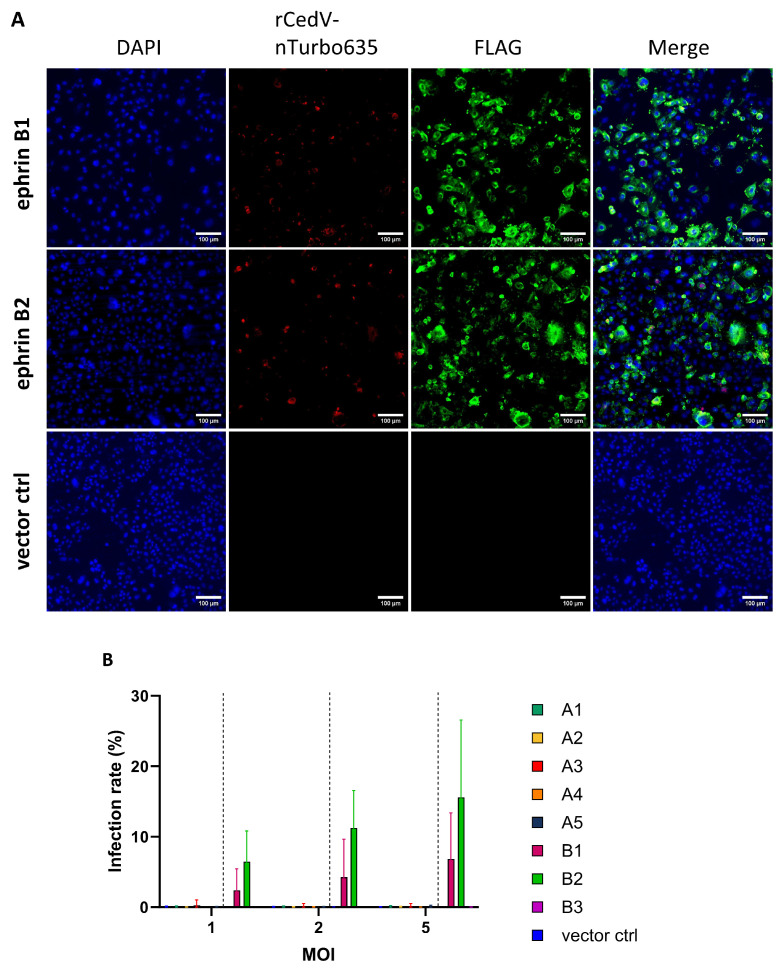

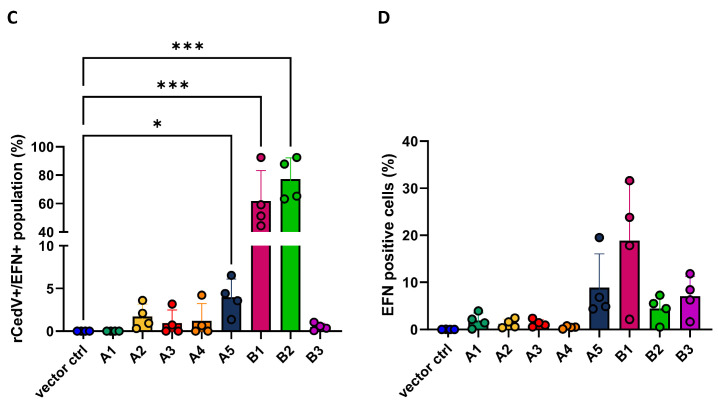
rCedV-nTurbo635 utilizes ERB ephrins B1, B2 and A5 for cell entry. (**A**) CHO-K1 cells expressing FLAG-tagged ephrins were stained with an Alexa488 fluorophore to monitor their expression. rCedV-nTurbo635 was visualized in the Cy3 channel. Representative pictures were taken from three independent experiments. Images were processed in ImageJ/Fiji. Scale bar: 100 μm. (**B**,**C**) High-content microscopy (**B**) and flow cytometry (**C**) analysis of rCedV-nTurbo635 entry. (**B**) CHO-K1 cells stably expressing ERB ephrins were grown in 96-well plates, re-transfected with plasmids encoding for A ephrins and infected with rCedV-nTurbo635 at MOI 1, 2 and 5. At 24 h post infection (p.i.), high-content imaging was employed to quantify the infection rate. Graph shows the median and range from three independent experiments in technical duplicates. Statistical test: Kruskal–Wallis without Dunn’s correction. (**C**) CHO-K1 cells stably expressing ERB ephrins were re-transfected with their respective ephrins and infected with rCedV-nTurbo635 (MOI 5) at 24 h post transfection. Graph shows the infection rate of the ephrin positive population 48 h p.i. (**D**) Quantification of FLAG-tagged ephrins by flow cytometry. Data show the mean ±SD from four independent experiments capturing 30,000 events each. (**B**,**C**) *p* < 0.05 (*) and *p* < 0.001 (***). Statistical test: Kruskal–Wallis without Dunn’s correction.

To validate our data with a live virus, a recombinant CedV expressing nucleus-localized Turbo635 fluorescent protein (rCedV-nTurbo635) was generated. Through using this virus, we could easily detect and quantify the percentage of infected cells. Further, CHO-K1 cells stably expressing ERB ephrins (CHO-EFN) were established for robust measurement. The expression of ERB ephrins was evaluated by Western blot and was also confirmed by immunofluorescence (Figure S2A–C and Figure 2A). Expression levels of A and B ephrins differed noticeably. Generally, the expression levels of B ephrins (except B3) were much higher than A ephrins (except A5) (Figure S2A–C). To visualize rCedV-nTurbo635 entry, FLAG-tagged ephrins were stained with an antibody conjugated with Alexa Fluor 488, and the virus entry was evaluated by monitoring the fluorescence of Turbo635 protein. Consistent with CedVpp data, rCedV-nTurbo635 entry could only be observed in cells expressing ERB ephrins B1 and B2 (Figure 2A). In order to analyze virus entry with large cell numbers, we established a protocol for automated image analysis using the CellInsight™ CX7 platform (Figure S2D). Briefly, CHO-K1 cells stably expressing ERB ephrins were infected with rCedV-nTurbo635 at different MOIs (1, 2 and 5), and rCedV-nTurbo635 entry was analyzed with automated microscopy. In agreement with the pseudovirus data, we detected remarkable virus entry only in CHO-EFNB1 and CHO-EFNB2 cells at all MOIs (Figure 2B and Figure S2E,F). The infection rate showed a clear dose dependency, with the highest values observed in ephrin B2 expressing cells. None of the A ephrin expressing cells supported rCedV-nTurbo635 entry, irrespective of the MOI used (Figure 2B and Figure S2E,F).

Finally, we assessed the infection rate by flow cytometry to exclude the effect of the variable expression levels of ephrins. In this assay, the infection rate was quantified at 48 h post infection (p.i.) since human ephrin A2- and A5-mediated CedV entry could only be observed 48 h p.i., as has been reported [67]. A gating strategy was established to allow gating of the different populations of interest (Figure S3). In accordance with the microscopy data (Figure 2B), both ephrin B1 and B2 expression can mediate rCedV-nTurbo635 entry (Figure 2C). In line with previous Western blot data (Figure S2A,B), the percentages of B ephrin-positive cells were generally higher than the ephrin A class, except ephrin A5 (Figure 2D). The expression level of ephrin A5 was comparable to ephrin B1 or B2 (Figure 2D). Interestingly, we also observed a low yet significant degree of ephrin A5-mediated entry at 48 h p.i. (Figure 2C). Combining our data of immunofluorescence, high-content microscopy and flow cytometry, we concluded that rCedV-nTurbo635 entry into ERB cells is mainly mediated by ephrin B1 and B2 and, to a lesser extent, by ephrin A5.

### 3.3. Ephrin B2 Knockdown Significantly Impairs rCedV-nTurbo635 Entry into ERB Cells

**Figure 3:**
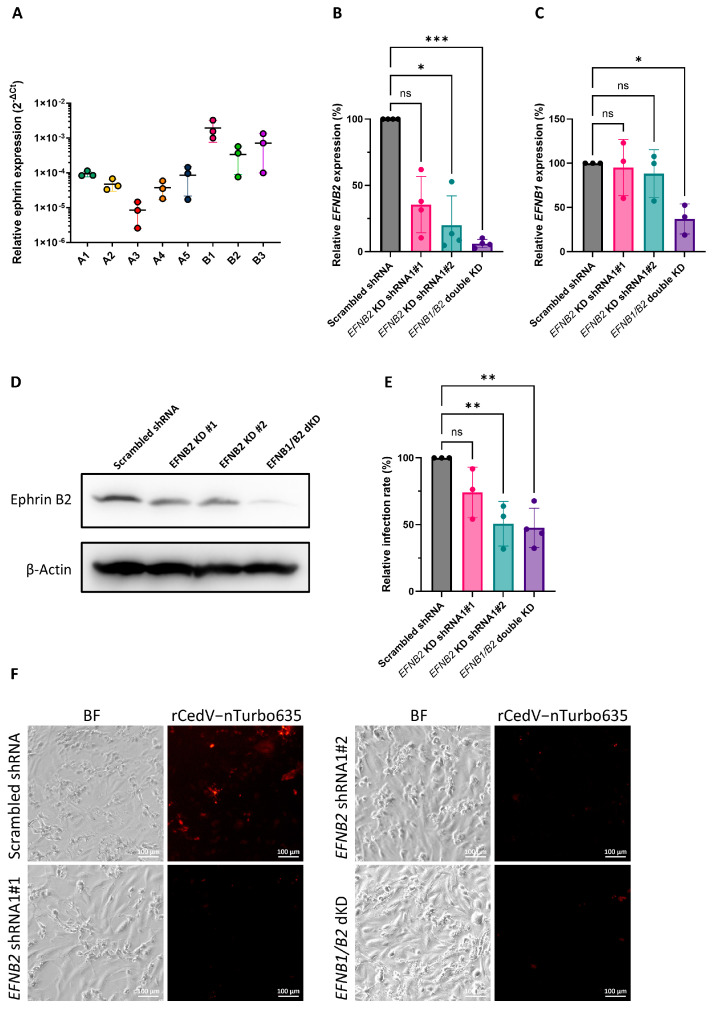
Ephrin B2 knockdown significantly impairs rCedV-nTurbo635 entry into ERB cells. (**A**) qPCR analysis of the ephrin expression in RaNep cells. The expression levels were normalized to the level of housekeeping gene EEF1A1. The data represent the mean ±SEM from three independent experiments. (**B**,**C**) qPCR analysis of the ephrin B2 (**B**) and ephrin B1 (**C**) expression in RaNep knockdown cells. The relative expression levels were normalized to the respective EFN expression level in the scrambled shRNA controls. The data show the mean± SD from at least three independent experiments. Statistical test: Kruskal–Wallis without Dunn’s correction; *p* < 0.05 (*) and *p* < 0.001 (***). (**D**) Western blot analysis of the ephrin B2 expression in RaNep knockdown cells. Representative Images were acquired from three independent experiments. (**E**) rCedV-nTurbo635 entry into RaNep cells with the knockdown of ephrin B2 or ephrin B1/B2. The ephrin knockdown cells were infected with rCedV-nTurbo635 at MOI = 1. Cell were then fixed at 48 h p.i. for flow cytometry analysis. Statistical test: one-way ANOVA corrected for multiple comparisons (Dunnett’s correction); *p* < 0.01 (**) and *p* > 0.05 (ns). (**F**) Fluorescent microscopy analysis of the rCedV-nTurbo635 entry in the RaNep scrambled shRNA control cells and the ephrin B2 and the ephrin B1/B2 KD cells. Representative Images were taken from two independent experiments. Scale bar: 100 μm.

To validate ERB ephrins B1 and B2 as the major entry receptors for CedV in ERB cells, we generated an immortalized cell line from *Rousettus aegyptiacus* nose epithelium and named it RaNep. To begin with, we evaluated the expression of endogenous ephrins in these cells by qPCR (Figure 3A). The expression levels of B ephrins were generally higher than A ephrins. Among all ephrins, ephrin B1 exhibited the highest expression level, while ephrin A3 showed the lowest (Figure 3A). Similar expression patterns were also observed in ERB lung endothelia-like cells (Figure S4). To elucidate the contribution of ERB ephrin B1 and B2 to rCedV-nTurbo635 entry, RaNep cells with ephrin B2 single knockdown (KD) and ephrin B1 + B2 double KD (dKD) cells were generated using the lentiviral shRNA system. We performed species identification with the cytochrome c oxidase subunit I (COI) barcoding to exclude carry over contamination of HEK293T cells and other cell lines maintained in the lab (Figure S5). Further, the knockdown efficiency was assessed by qPCR analysis. Both ephrin B2 shRNA1 clone#2 cells and ephrin B1/B2 dKD cells exhibited an efficient reduction in ephrin B2 expression with only 20% and 6% residual expression compared to the scrambled shRNA control (Figure 3B). The dKD cells additionally showed a 63% reduction in ephrin B1 expression, whereas ephrin B1 remained unaltered in the single ephrin B2 KD clone#2 cells (Figure 3C). Western blot analysis further confirmed the drastic reduction in ephrin B2 protein level in the dKD cells (Figure 3D). Subsequently, the cells were infected with rCedV-nTurbo635, and infection rates were quantified using flow cytometry. The infection rates in both the ephrin B2 KD clone#2 and the ephrin B1/B2 dKD cells were significantly reduced compared to the control cells (Figure 3E). As expected, the infection rate in ephrin B2 KD clone#1 cells with inefficient KD did not differ significantly from that in the control cells (Figure 3C,E). Interestingly, the infection rate in ephrin B1/B2 dKD cells did not differ significantly from that in the ephrin B2 single KD cells (Figure 3E), supporting the importance of endogenous ephrin B2 in the rCedV-nTurbo635 entry into ERB cells. The reduced entry of rCedV-nTurbo635 due to ephrin B2 KD was further confirmed by fluorescent microscopy (Figure 3F). All in all, we conclude that CedV entry into ERB epithelial cells is reduced by ephrin B2 knockdown.

## 4. Discussion

The Egyptian Rousette bat (*Rousettus aegyptiacus*, ERB) is well established as the reservoir host of the Marburg virus [83,84], and it is also considered as the reservoir of Sosuga virus, a novel member in the virus family *Paramyxoviridae* that causes severe febrile disease in humans [85,86]. Recently, a growing number of henipaviruses and related viruses associated with this bat species have been detected in Africa [30,32,87], supporting ERB as an important host of henipaviruses [30]. Phylogenetic analysis has demonstrated that multiple henipaviruses identified in ERB are closely related to CedV, a bat-borne henipavirus originally isolated from pteropid bats [30,37]. Intriguingly, experimental challenge of ERB with NiV or CedV has revealed that ERB are unable to support productive replication of both viruses [88,89]. Thus, it is worthwhile to investigate whether CedV can enter ERB cells and whether subsequent processes, such as genome replication and host immune responses, restrict the cross-species transmission of CedV.

In this study, we aimed to investigate whether CedV can enter ERB cells and which receptors mediate the CedV entry into ERB cells. To answer these questions, we generated CedV pseudotyped particles (CedVpp) based on a lentiviral system. This approach can also be utilized for investigating the entry and neutralization of other newly identified henipaviruses in the future. In combination with rCedV-nTurbo635 data, we demonstrated that ERB ephrin B1, B2 and, likely, ephrin A5 mediate CedV entry. However, only minor ephrin A5-mediated entry was observed at 48 h p.i., indicating against the importance of endogenous ERB ephrin A5 in CedV entry. Ephrin protein alignment between ERB and *Pteropus alecto*, from which CedV was first isolated, revealed that the G-H loops of ephrin B1, B2 and A5 from these two species are identical (Figure S1). Thus, we postulate that CedV may use the same receptors to enter cells of its natural host. In contrast to human receptor usage [67], we did not observe ERB ephrin A2-mediated CedV entry in any of our assays, although the G-H loops of ERB and human ephrin A2 sequences were identical. The binding affinity of human ephrins A2 and A5 to CedV G is around 500-fold lower than for ephrins B1 and B2 [67]. Thus, the lack of ephrin A2-mediated entry could be due to the low binding affinity of CedV G to ERB ephrin A2. Another possible reason is that the expression level of ERB ephrin A2 is lower than other ephrins. To exclude the effect of low expression level of ephrin A2, we gated on the cells highly expressing ephrin A2 in flow cytometry, but we still did not observe rCedV-nTurbo635 entry. Hence, it is likely that ERB ephrin A2 cannot mediate CedV entry. To validate our findings on CedV entry mediated by ERB ephrin B1 and B2, we also generated ERB cells with endogenous ephrin B1/B2 KD. CedV infection was significantly impaired by ephrin B2 KD, demonstrating the importance of endogenous ephrin B2 in mediating CedV entry into ERB epithelial cells. Unfortunately, we did not succeed in generating cells with efficient ephrin B1 single KD. In future research, we will put more effort into generating ephrin B1 single KD cells to uncover the contribution of endogenous ephrin B1 in mediating CedV entry into ERB cells.

HeV and NiV G proteins stabilize their contact with the ephrin B3 G-H-loop (E119-W125) [59,90] by non-covalent pi interactions between the aromatic rings of their tyrosine residues (Y581) and the aromatic ring of ephrin B3 (Y120) [67]. However, CedV G protein has an asparagine at the equivalent position, leading to abrogation of pi-stacking and, hence, unstable interaction with ephrin B3. However, in silico analyses suggest substituting the CedV G asparagine residue at position 602 with a tyrosine could be sufficient for ephrin B3 usage [67]. Generation of CedVpp containing a mutated form of the G protein and investigation of its entry may help us to uncover the different receptor preferences by CedV.

The entry receptors for HeV and NiV, ephrin B2 and B3, are highly conserved across vertebrate species [91]. Protein sequence alignment of ephrin B2 and B3 from multiple species has revealed a high degree of conservation among human, horse, pig, cat, dog, mouse, *Pteropus alecto* and *P. vampyrus* bats, respectively [53]. In vitro expression of ephrin B2 or B3 from all these species is able to support HeV and NiV infections [53]. Consistently, many animals, including guinea pigs [92], hamsters [93], ferrets [94], dogs [95] and cats [96], have been demonstrated to be susceptible to HeV and or NiV upon natural infection or experimental challenge. Therefore, it is proposed that the wide host range of henipaviruses is attributed to the high conservation of ephrins. Here, we discovered that ERB ephrin B1, B2 and, likely, A5 can mediate CedV entry; however, ERB appears to be resistant to CedV infection upon intranasal challenge [89]. In a similar direction, mice are resistant to NiV infection, although the expression of the highly conserved murine ephrin B2 and B3 leads to NiV entry in vitro [53,93,97]. Therefore, restrictive mechanisms operating at the post-entry step limit the establishment of successful infection of henipaviruses, likely representing barriers to their cross-species transmission. Intriguingly, mice deficient of IFN-I receptor (*Ifnar*^−/−^) develop fatal encephalitis upon NiV and HeV infection [98], suggesting the importance of IFN-I signaling in controlling henipavirus infection. Considering ERB as an important host harboring henipa-related viruses, henipavirus infection in ERB cells could be employed as a model to investigate the mechanisms of antagonizing IFN signaling by different henipaviruses, where the aim is to shed light on the molecular determinants of cross-species transmission.

## Figures and Tables

**Table 1:** The shRNA oligos used for knockdown.

shRNA	Target	Targeting Sequence
shRNA1	ERB ephrin B2	AGGAGACAAATTGGATATTAT
shRNA2	ERB ephrin B2	GCCGGACATTCTGGGAATAAT
shRNA3	ERB ephrin B2	TGTTGGCCAGTATGAATATTA
shRNA4	ERB ephrin B1	TGGTCATCTACCCAAAGATTG
shRNA5	ERB ephrin B1	AGCACCATGATTACTACATTA
shRNA6	ERB ephrin B1	TGTGCTGGTCACCTGCAATAA

**Table 2:** (q)PCR primers.

Target	Primer Sequence	Amplicon Size
ERB ephrin A1	F: TGGGCAAGGAGTTCAAAGAG R: AACCTCAAGCACCTGTCTTC	97 bp
ERB ephrin A2	F: GCTCTTCACGCCCTTCTC R: GCCGCACGTAGACCTTC	122 bp
ERB ephrin A3	F: CATGCGGTGTACTGGAACAG R: TTGTAGTGCGGGCAGTAAATATC	104 bp
ERB ephrin A4	F: AAGGAGAGCAAGTCGGAGTC R: TCAGAGAACTCGCAGGAGTC	147 bp
ERB ephrin A5	F: TCTACTGGAACAGCAGCAAC R: GGACATAACGCTCGGTCTTATC	135 bp
ERB ephrin B1	F: GACTGTGAACCAGGAAGAGAAG R: CCGTCAGGAAGATGATGATGAG	152 bp
ERB ephrin B2	F: TGGTACTATACCCACAGATAGGAG R: TTTGGCACAGTTGAGGAGAG	167 bp
ERB ephrin B3	F: GAGAAGGTGAGTGGTGACTATG R: GTAGATGTTTGGAGGACTCTGG	90 bp
Egyptian Rousette bat CO1	F: TTC TAC CCC CAT CTT TTC TTC TTC TAT TAG R: GGA TAG GGC TGG TGG TTT TAT ATT AAT AAT	250 bp
Tamarin monkey CO1	F: TCA ACT TCA TCA CCA CAA TCA TTA ACA TAA R: CTC ATA CGA TAA ACC CTA AAA ATC CGA TAG	400 bp

## Data Availability

All original images of western blotting and immunofluorescence have been deposited to Zenodo (10.5281/zenodo.15172463).

## Supplementary Materials

The following supporting information can be downloaded at: https://www.mdpi.com/article/10.3390/v17040573/s1, Figure S1: Protein alignment of ephrins from human, Egyptian Rousette bat and *Pteropus alecto*. G-H-loop (purple) was originally defined as the region from E119-S128 in EFNB3, which is identical in human, ERB and *P. alecto* EFNB3 [59]. Protein alignment was performed with Geneious Prime (Version 2021.0.1) and visualized with ESPript 3.0 [99] using the protein sequences from NCBI Genbank (Reference genomes: *P. alecto*: GCF_000325575.1, ERB: GCF_001466805.2, human: GCF_000001405.40); Figure S2: (**A**,**B**) Western blot analysis of the FLAG-tagged ERB ephrins. CHO-K1 cells stably expressing FLAG-tagged ephrins were re-transfected with plasmids encoding for A ephrins (**A**) or B ephrins (**B**). Then, 24 h later, cell lysates were collected for Western blot analysis. The expression of ephrins was evaluated with an anti-FLAG antibody, and β-Actin serves as the loading control. (**C**) Immunofluorescence microscopy of cells expressing FLAG-tagged ephrins with rCedV-nTurbo635 infection. Pictures are representative of three independent experiments. Images were edited in ImageJ/Fiji. Image processing was standardized in the Cy3 channel for all ephrins, whereas brightness adjustments in the FITC channel were conducted for A ephrins plus B3 and B1/B2 separately. Scale bar: 100 µm. (**D**) Details about the high-content imaging microscopy. The blue mask identified DAPI-stained nuclei as primary objects, which were subsequently filtered by size, shape and brightness to receive the valid objects. In the red channel, a green mask recognized fluorescence above background levels as a positive event, but only if the fluorescence was directly adjacent to a valid primary object. For analysis of the infection rate, the parameter “Average intensity of channel 2” was used, and only “highly” positive events were interpreted as infected cells. Thresholds for fluorescent intensities were determined using rCedV-nTurbo635-inoculated pcDNAflag expressing CHO-K1 cells as the negative control and rCedV-nTurbo635-infected BHK-21 as the positive control. (**E**,**F**) High-content microscopy analysis of rCedV-nTurbo635 entry. CHO-K1 cells stably expressing ERB ephrins were cultivated in a 96-well plate and infected with rCedV-nTurbo635 at MOI 5. At 24 h p.i., high-content image analysis using the Thermo Scientific™CellInsight™ CX7 was employed to quantify infection rate. Graphs represent three independent experiments in technical duplicates (median and range). *p* < 0.05 (*) and *p* < 0.01 (**). Statistical test: Kruskal–Wallis without Dunn’s correction; Figure S3: Flow cytometry gating strategy for analyzing rCedV-nTurbo635 entry. CHO-K1 cells stably expressing FLAG-tagged ephrins were infected with rCedV-nTurbo635 for 48 h and then stained with an anti-FLAG antibody, which was followed by a BV421-conjugated secondary antibody. Cells were first gated to exclude duplicates and dead cells. Additionally, two gates were applied: BV421 positive cells were considered ephrin positive and CF594 positive cells were considered infected; Figure S4: qPCR analysis of ephrin expression in ERB lung endothelial-like cells. The expression levels were normalized to the housekeeping gene *EEF1A1*. The data represent the mean ± SEM from four independent experiments. Figure S5: Species identification of ephrin knockdown RaNep cells. The cytochrome c oxidase I (COI) barcoding PCR was performed to exclude contamination with other cell lines. The positive controls were as follows: Human: 390 bp, pig: 460 bp, mouse: 150 bp, Egyptian Rousette bat: 250 bp and Tamarin monkey: 400 bp.
