# Supplementary figures and images for: Ephrin B1 and B2 Mediate Cedar Virus Entry into Egyptian Fruit Bat Cells
Source: Viruses. 2025 Apr 16;17(4):573. doi: 10.3390/v17040573 (PMC12030902; doi:10.3390/v17040573)

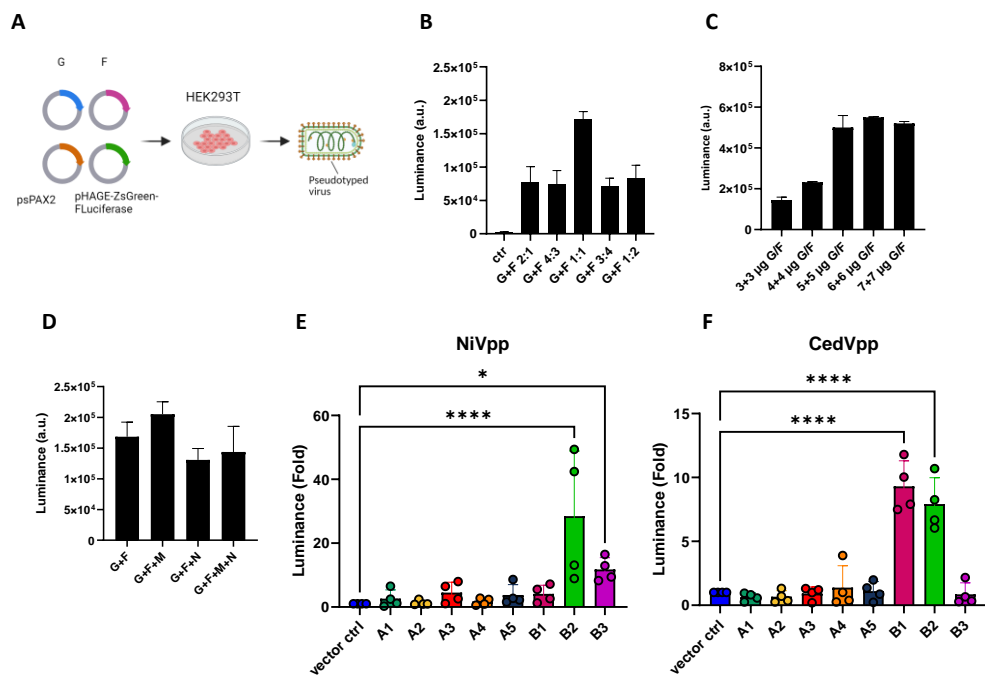

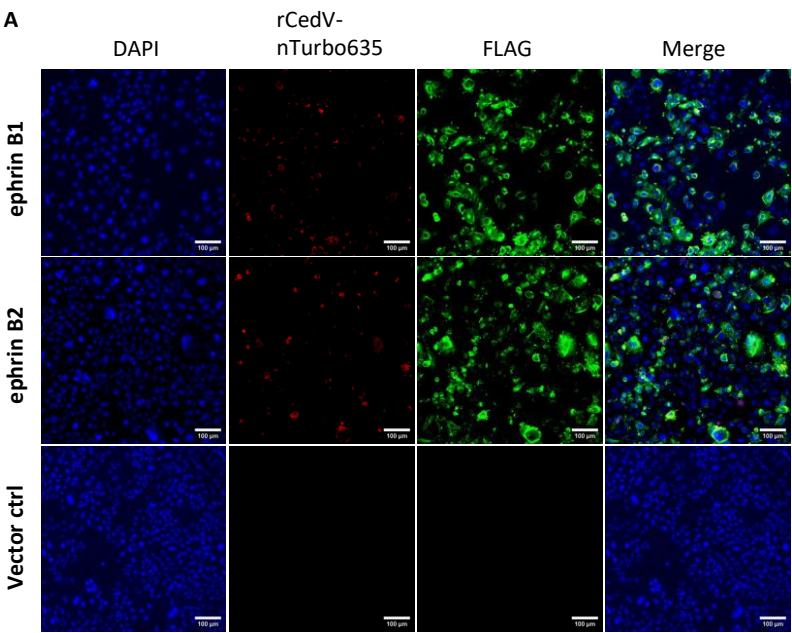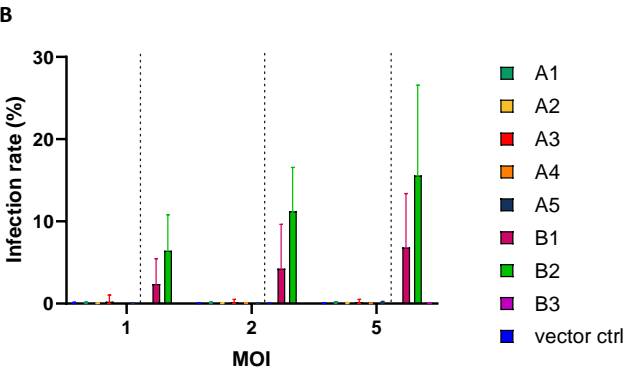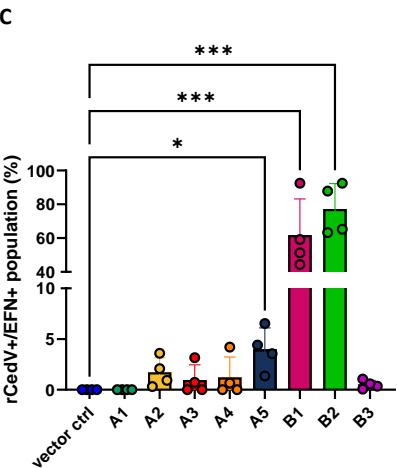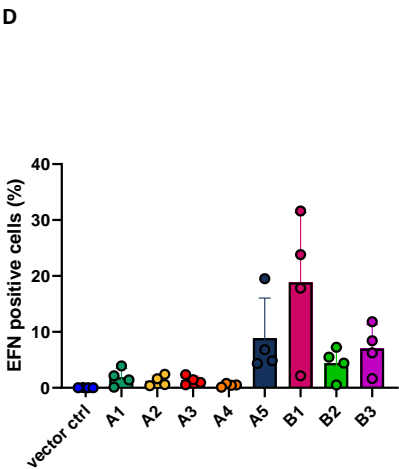

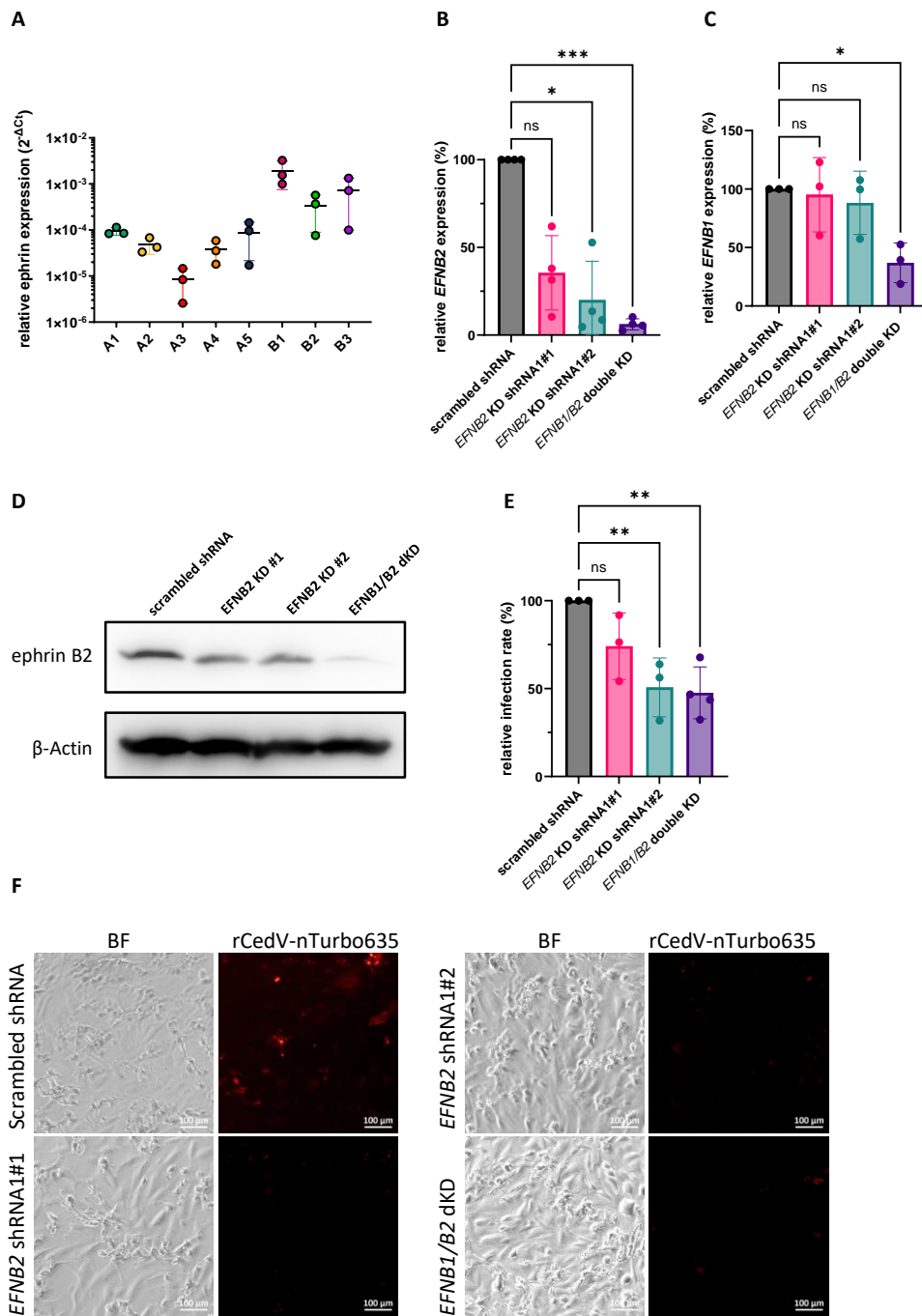

Figure S1

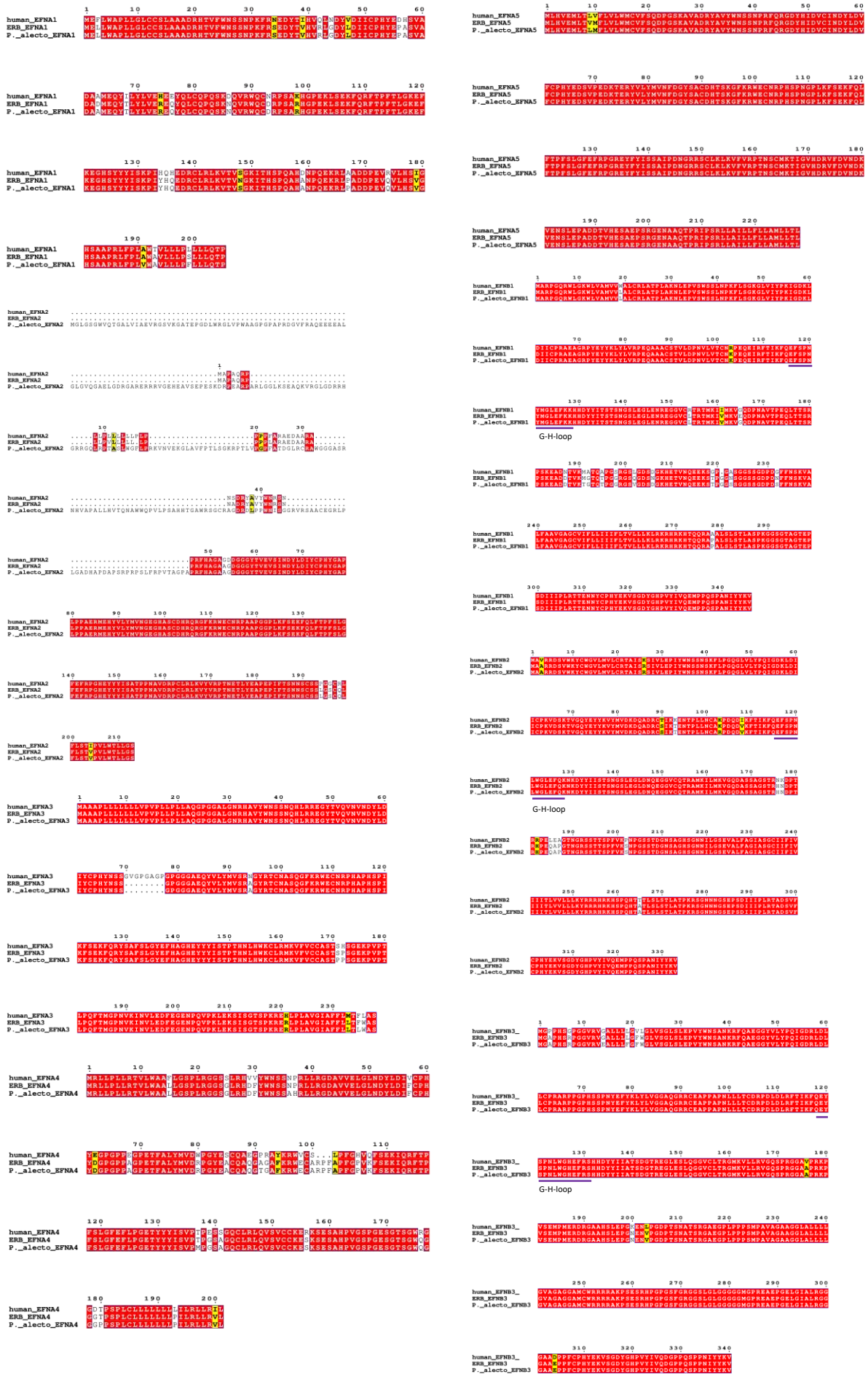

Figure S2

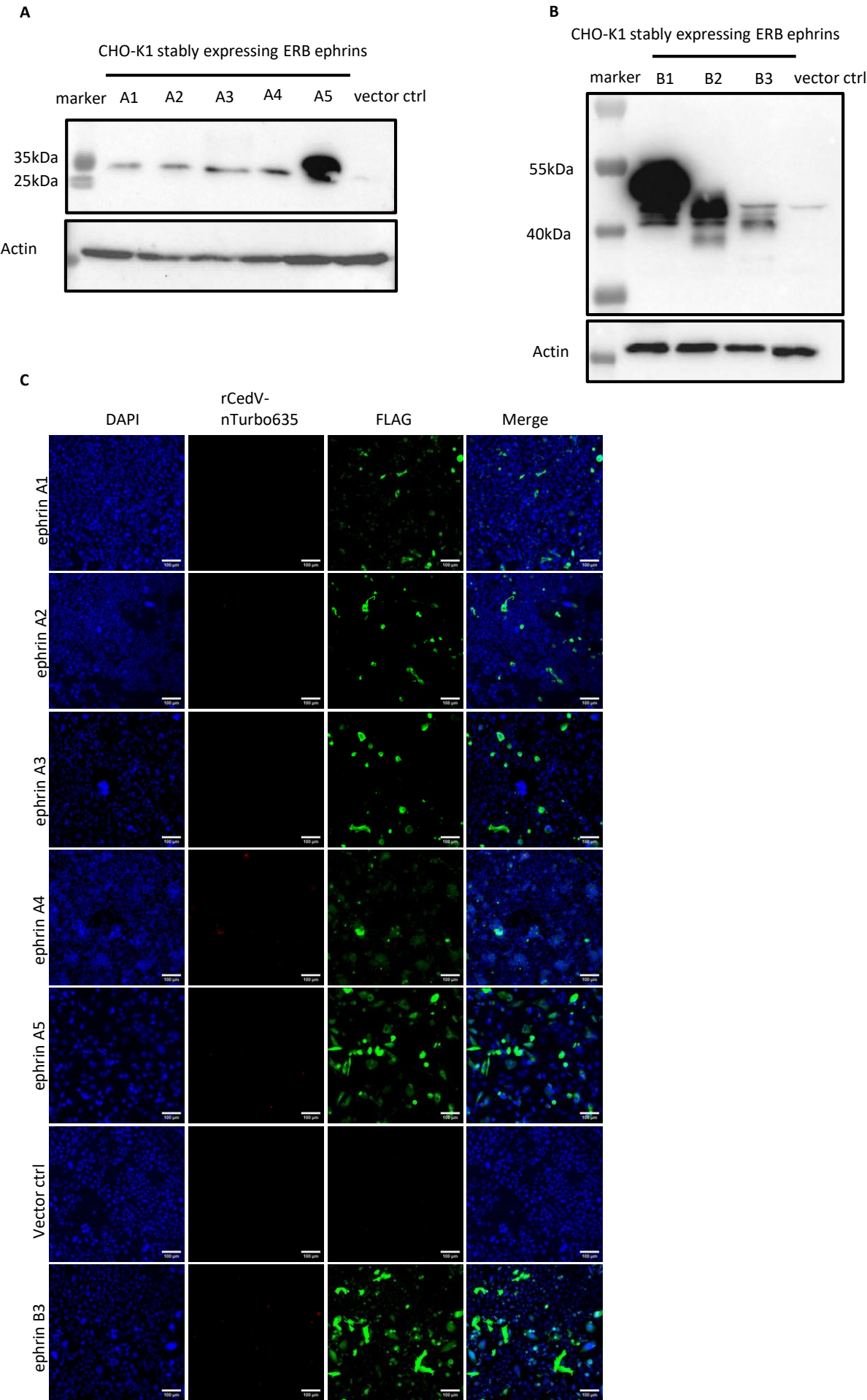

Figure S2

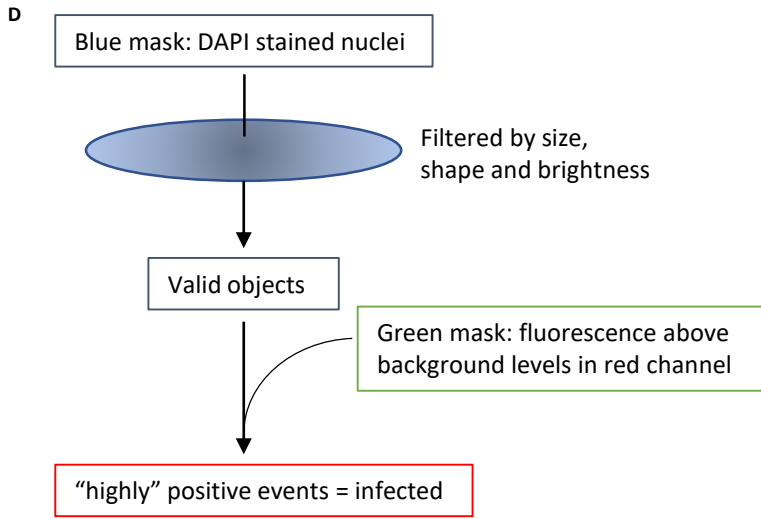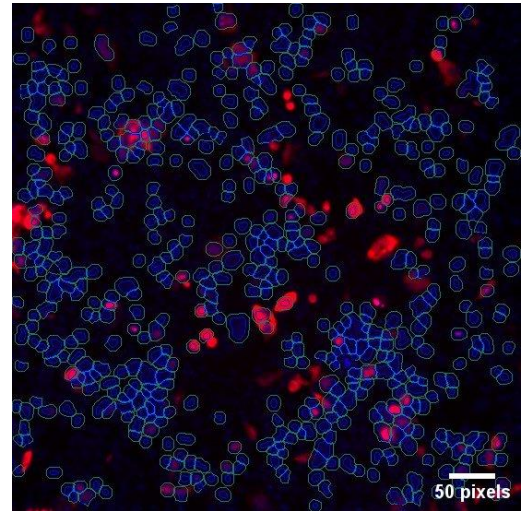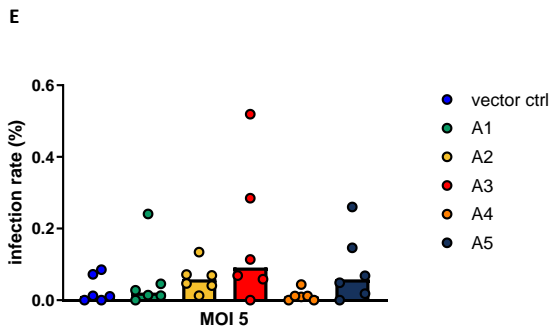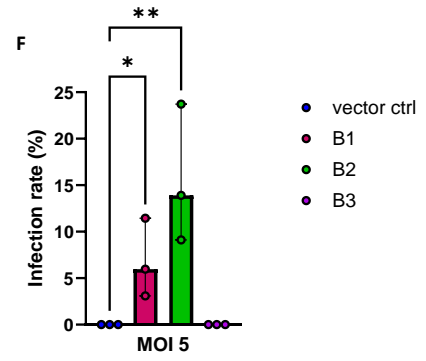

Figure S3

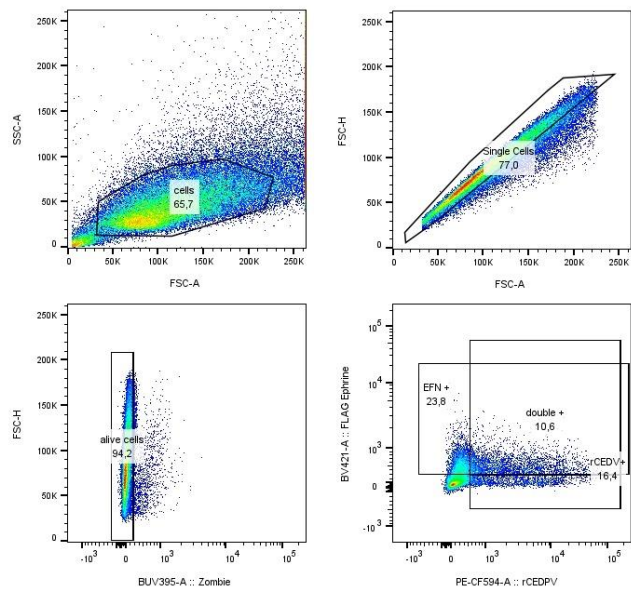

Figure S4

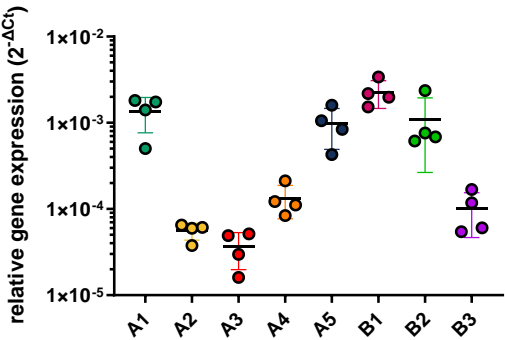

Figure S5

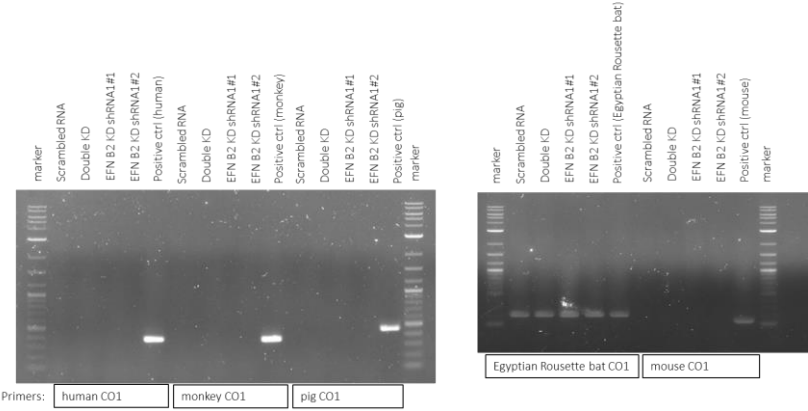

Supplement: Supplementary file 1 [file viruses-17-00573-s001.zip › viruses-3464343-supplementary.pdf]
